# Barriers and facilitators to HIV pre-exposure prophylaxis uptake among transgender women in Colombia: A qualitative analysis using the COM-B model

**DOI:** 10.1371/journal.pgph.0001395

**Published:** 2023-09-27

**Authors:** Maria Camila-Bolívar, Sheilla Andrea Gomez-Peñaloza, Pilar Camargo-Plazas, María del Pilar Peralta-Ardila, Héctor F. Mueses-Marín, Beatriz Alvarado-Llano, Jorge L. Martínez-Cajas

**Affiliations:** 1 Department of Public Health and Epidemiology, Pontificia Universidad Javeriana Cali, Cali, Colombia; 2 Corporación de Lucha Contra el Sida, Cali, Colombia; 3 School of Nursing, Queen’s University, Kingston, Ontario, Canada; 4 Public Health Science, Queens University, Kingston, Ontario, Canada; 5 Division of Infectious Diseases, Department of Medicine, Queens University, Kingston, Ontario, Canada; University of Mississippi Medical Center / John D. Bower School of Population Health, UNITED STATES

## Abstract

Transgender women [TGW] in Colombia are disproportionately affected by HIV due to their low sociodemographic conditions, varied risk behaviours, difficulty accessing health services, and discrimination. Offering pre-exposure prophylaxis [PrEP] as part of a combination of prevention strategies is an appropriate option for this population to reduce their risk of HIV infection. However, little is known about how to implement a PrEP program for TGW in Colombia. Between June and October 2020, we conducted individual interviews with 16 TGW from four different cities in Colombia. The interviews assessed contextual influences, knowledge, skills, perceptions, and beliefs. We used qualitative thematic analysis to identify themes and the Capability, Opportunity, Motivation, and Behavior framework to further delineate barriers and possible interventions. After delineating the main themes across the three subdomains of the model, nine barriers were identified: one related to capability, knowledge, and perception of PrEP; six related to opportunity, which includes, family relations, sexual work environment, stable partner relations, interactions with healthcare workers, health service provision, and community interactions and opportunities; and two related to motivation, mental health, and concerns about medication side effects. Mapping barriers with interventions generated the following intervention functions: education, training, enablement, and environmental structure; and the following policy functions: communication/marketing, legislation, and changes in service provision. Examples of possible interventions are presented and discussed.

## Introduction

Transgender women [TGW] are a high-risk population for HIV infection. HIV prevalence in the TGW population has been estimated to be 37 times higher than in the general adult population and two to three times higher than in men who have sex with men [MSM] [1]. In Colombia, the prevalence of HIV among TGW remains the highest among all population groups, with a reported prevalence of 21.4% in 2020 compare to Men who have sex with Men with a reported prevalence of 18,0% [2]. As in other low- and middle-income countries, the vulnerability of TGW in Colombia is related to their experiences of systemic discrimination [e.g., limited access to education and employment], involvement in sex work for economic survival, and gender reaffirmation as well as high levels of depression, use or abuse of substances, and violence [3, 4]. TGW living with HIV show low rates of retention in care, which increases their risk of disease progression and perpetuates HIV transmission [5]. TGW have limited access to HIV prevention interventions [6, 7], including awareness, uptake, and adherence to HIV pre-exposure prophylaxis [PrEP] [8, 9], an HIV-prevention method recommended by the World Health Organization to help eliminate HIV [4].

Expanded access and adoption of PrEP are urgently needed in Latin America to curb the epidemic in the TGW population and address the many gaps across settings and populations [10]. Brazil was the first country to offer free access to PrEP for populations at substantial risk of HIV infection [11] and has led studies on the adaptation and innovation of PrEP delivery. In 2018, Mexico joined the ImPrEP pilot project, along with Brazil and Peru, in an attempt to implement a public policy of universal access to PrEP [12]. Although the overall experience in Latin American countries has shown that PrEP is feasible and safe, PrEP awareness, retention, and uptake among TGW remain low [12]. In 2019, Colombia approved the use of tenofovir disoproxil fumarate/emtricitabine for HIV prevention and conducted one demonstration project, which recruited TGW and individuals from other high-priority groups [13]. At the end of 2021, Colombia issued a policy for PrEP coverage in its healthcare system, and in 2023, the Ministry of Health released implementation guidelines for physicians to prescribe PrEP [14]. Results from the demonstration project in Colombia have not been published, thus data on the acceptability and adherence of TGW to PrEP in Colombia remains unknown. Situational analyses in Colombia around PrEP implementation strategies remain limited to the work conducted by our group through the project PrEP-COL.

The results presented here are part of a larger project [PrEP-COL] that assesses factors that influence the implementation of PrEP in Colombia [15]. PrEP-COL uses a mixed-method design to collect information on barriers and facilitators for PrEP adoption in HIV clinics, adoption among healthcare providers, and uptake in populations at high risk of HIV and delineates strategies for PrEP implementation in Colombia [16]. PrEP-COL remains the only country-wide study generating data on the determinants of PrEP adoption/uptake in Colombia. We recently reported that healthcare providers recommended that personal [i.e., lack of knowledge and negative attitudes], contextual [i.e., stigma and lack of funding], and organizational [i.e., available resources] be addressed to scale up PrEP provision nationally [16]. Preliminary analysis in a sample of MSM identified the importance of positive attitudes, PrEP stigma, and PrEP skills for the intention to use PrEP [17]. Survey data from 158 TGW in three large Colombian cities revealed that only 26% of participants were aware of PrEP, but 67% were willing to use PrEP if offered at no charge [18]. In this sample, a lack of adequate housing, young age [<24 years old], depressive symptoms, and negative attitudes/motivations toward PrEP decreased their willingness to use PrEP [17].

In this paper, we report findings based on interview data from the PrEP-COL study with TGW, which took place simultaneously with the survey and expands and deepens our understanding of the subject. The main objectives of this study were to identify barriers and facilitators for PrEP uptake [Objective 1] and to use this knowledge to formulate behavioural interventions and policy strategies to address these barriers in this population [Objective 2].

## Method

### Ethics statement

The Research Ethics Boards of both the Corporación de Lucha contra el SIDA [approval certificate no. 034 of May 16, 2018] and Queen’s University [DMED-2326-20] approved this study. The consent was obtained in written form.

#### Conceptual framework

To achieve our objectives, we used the Capability, Opportunity, Motivation, and Behavior [COM-B] model, a behavioural change framework that recognizes that behaviour [in this case, PrEP uptake] is influenced by an individual’s knowledge and skills to perform such behaviour, the external and physical factors that either promote or block that behaviour, and the basic drives and reflections that can affect their willingness to perform a certain behaviour [19]. We postulated that factors related to PrEP acceptance among TGW could be mapped into all domains of the COM-B framework. As depicted in **Table 1**, studies with TGW demonstrated the relevance of various factors on PrEP use that can be mapped in the COM-B. Factors related to the capability domain include HIV prevention knowledge, and skills; factors related to opportunity dimensions include societal influences, resources, interactions with healthcare systems, family, and peers, and HIV stigma; and motivational factors include beliefs, intentions, concerns, mental health states, and habits [8, 9, 20, 21]. The COM-B relies on the delineation of explanatory factors that influence a particular behaviour on multiple levels [i.e., individual, interpersonal, and contextual], which helps us to identify barriers and facilitators, and a structured framework for intervention design [Objective 1]. COM-B lies at the centre of the Behavior Change Wheel [BCW], a toolkit that assists in the selection and design of behavioural interventions and strategies to address identified barriers [19]. By categorizing barriers and facilitators into capability, opportunity, and motivational factors, the COM-B and the BCW will help us to delineate specific areas for intervention [Objective 2].

**Table 1:** Examples of how the COM-B model could be applied to the intention of using PrEP in TGW*.

Capability	Opportunity	Motivation
**Psychological: knowledge, memory attention**	**Physical aspects of the environment: physical and environmental resources**	**Reflexive: beliefs about capabilities, consequences, intentions**
Knowledge about HIV prevention, e.g., using condoms	Access to and cost of medications	Beliefs and perceptions about PrEP, interactions with hormones, fear of side effects
Knowledge and awareness about PrEP	Offer of services for prevention and monitoring of PrEP	Perceived risk of HIV
**Physical: skills and abilities acquired through practice**	**Social aspects: influence of others, norms, support**	**Automatic: needs and mental health state**
Ability to communicate with healthcare professionals	Stigma and discrimination: HIV and PrEP related, at all levels, family, community, and health care providers	Stigma, depression, anxiety, and other mental states
		Use of hormones
Condom use negotiation skills	Limited social opportunities, low education, sex work, low levels of income	Compensation of risk- decreased condom uses if using PrEP [less frequent in TGW than in MSM]
Medication adherence skills	Sexual and relationship partners	Habits: drug and alcohol use

*Data in the table summarizes published literature reviews [1, 8, 9, 12, 20]

### Research design

For this qualitative study, we employed a descriptive scope to identify the barriers, facilitators, and preferences of TGW concerning their intention to use PrEP. The fieldwork was conducted from June to October 2020 and happened concurrently with the collection of survey data.

### Participants

A convenience sample of TGW was recruited by TGW community leaders working with local non-governmental organizations. The community leader identified potential participants and then connected them with researchers to take part in the study if interested. Individuals were eligible if they identified themselves as a Colombian transgender woman, were 18 years of age or older, and were not living with HIV. The exclusion criterion was being under the influence of psychoactive substances at the time of the interview. Participants consented to participate in the study and to the interview being audio recorded. The participants received Colombian $30,000 [approximately US$7] compensation for their time.

### Data collection techniques

Semi-structured interviews were conducted with a predetermined series of questions but with the flexibility to add other relevant questions as appropriate. Before starting the interview, participants were introduced to the basic concepts of PrEP by viewing a video or reading a defined statement when applicable. This allowed the interviewer to guide the questions on perceptions, willingness, facilitators, and barriers. The interview guide was created by the research team [PC, SG, BA] based on the bibliography collected during the background writing process [S1 Text]. The interview guide aimed to capture 1] experiences at the HIV-prevention level and those specifically related to PrEP, and for each of the experiences reported, 2] the influences at different levels [i.e., personal, interpersonal, and community], emphasizing those that could be conceptualized as a barrier or an opportunity. Two researchers with graduate training in sociology and experience in qualitative methods conducted the interviews. Only one interview was conducted with each participant, and the interviews lasted from 45 to 120 minutes. Due to COVID-19 restrictions that prevented travel and in-person interactions with participants, five interviews were conducted via Zoom and 11 by phone. We ensured the trustworthiness of the study using the strategies presented in **Table 2** [22, 23].

**Table 2:** Trustworthiness criteria applied in this work.

Criteria	Strategies used
**Credibility:**	Accomplished through the descriptions of demographic characteristics, the inclusion of the voices of all participants, and the comparison of our results with the results from the survey in TGW [24].
**Dependability:**	The experiences of our participants were unique, as they have not been part of other interventions. It is possible that the COVID-19 epidemic has affected some experiences but none of the narratives seem to report any major impact of COVID-19 in their perceptions and experiences, especially regarding the possibility of using PrEP.
**Conformability:**	Three people examined the data, two summarized the main findings, two coded the interviews, and two discussed the themes and narratives. Our findings were shared with TGW in focus groups and agreement with findings and narratives and themes were supported [25].
**Transferability**	Our data reflect the experiences of different transgender women living in Colombia. Notably, their experiences could be different than other TGW from small cities or different race/ethnicities; however, many of their experiences with HIV and PrEP seems to be consistent across studies and populations of TGW in different Latin-American contexts
**Authenticity**	The narratives here reflect the experiences of a diverse socioeconomic and age sample, from four different Colombian cities. Their experience reflects what has been found in other studies, such as the low awareness and high intention, and the low experience with PrEP in the sample.
**Rich rigor**	Our study uses a theoretical construct to delineate barriers facilitators and interventions, the context and the sample are appropriate to the study as the TGW population is at high risk of HIV.
**Significant contribution**	Our study provided a significant contribution by innovatively using a theoretical framework and providing a set of possible interventions needed to address the barriers identified.

### Analysis

The interviews were transcribed verbatim in Spanish by a specialized transcription company bound by a confidentiality agreement. MCB and MPP validated the audio and transcription accuracy. The transcriptions were processed using the data analysis software ATLAS.ti and a standardized codebook. The codebook was created by MCB and MPP and reviewed by three of the researchers [BA, SG, and PC] using a hybrid analysis: coding themes using a predetermined framework and adding themes as they emerged. The initial coding included seven major domains: 1] contextual influences, 2] knowledge, 3] perceptions and beliefs, 4] healthcare system, 5] social interaction, 6] skills, and 7] action plans. Subsequently, MB and MPP synthesized and consolidated the results obtained using the proposed domains. Four of the researchers [BA, PC, MCB, and SG] proceeded to prepare the results, consolidate the interpretations, discuss the information, and specify the conclusions of the study. The full analysis is accessible online [18]. For this report, the most important themes and narratives were selected and mapped onto the domains or components of the COM-B model and further shared with the rest of the team for review, after which interpretations of the results were refined. Finally, the identified barriers were mapped again using the BCW framework to identify interventions and policies [19]. This process followed the steps suggested by Michie et al. [19] and was completed by two of the authors [MCB, BEA] and reviewed for refinement by all other authors.

## Results

### Participants

Sixteen TGW aged 18 years or older consented to participate in the study. The participants were residents of the cities of Cali [n = 4], Medellin [n = 3], Bogota [n = 3], and Barranquilla [n = 6]. **Table 3** describes the socio-demographic profile of the participants. Participants came from diverse social backgrounds and with diverse experiences on PrEP, mostly with low previous awareness and low uptake.

**Table 3:** General characteristics of the participants, TGW from Colombia.

Age	Item	Percentage	No.
Level of schooling	Unfinished Bachelor’s degree	31%	5
	Bachelor’s degree completed	37%	6
	Technician	19%	3
	Student	12%	2
Occupation	Human Rights Activist	19%	3
	Stylist	25%	4
	Sex worker	44%	7
	Student	6%	1
	Saleswoman	6%	1
Type of affiliation to the Colombian health system	Subsidized	38%	6
	Contribution	31%	5
	No affiliation	31%	5
City of residence	Cali	25%	4
	Medellin	19%	3
	Bogota	19%	3
	Barranquilla	37%	6
PrEP awareness	Yes	31%	5
	No	69%	11
Willing to use PrEP	Yes	88%	14
	No	12%	2
Experience with PrEP	Yes	6%	1
	No	94%	15

### Main themes

We identified 13 major themes, which were mapped within the three main domains of the COM-B model [i.e., capability, opportunities, and motivations]. The themes according to the three COM-B categories are presented below with narrative excerpts from the interviews.

#### 1. Capability

This domain summarizes the experience of participants concerning HIV prevention knowledge and skills. The themes relate to knowledge and skills that influence their acceptability of PrEP, including continuing condom use, HIV testing, taking pills, and attending PrEP monitoring visits.

*1.1. HIV prevention knowledge and skills*. Participant narratives demonstrated knowledge about HIV and its prevention, including the effectiveness of consistent condom use, the importance of getting HIV tested routinely, and the risk associated with having sex with partners of unknown serostatus. This knowledge could facilitate PrEP uptake. Mariana [44 years old] mentioned, *"Prevention is not only using a condom but also looking at who you are going to be with; in my case*, *it means not being too promiscuous*.*"*

All participants used condoms as their main HIV prevention tool. They know that the efficacy of condoms is high when used correctly and that it is decreased if broken; most of them have high levels of confidence in their effectiveness, as in the case of Fernanda:

*I don’t know if I can say 100%*, *but I feel more confident when I use one*. *If I didn’t use one*, *I would be scared*, *but I always make sure it’s on correctly or that it doesn’t break … the condom has given me a lot of confidence*. *[Fernanda*, *50 years old*]

The participants have gained this knowledge through different means: interactions with community organizations and transgender leaders; learning from their own experiences, such as previous sexually transmitted diseases; sexual encounters with partners living with HIV; and sexual education at school.

Participants mentioned their capacity to negotiate condom use with partners, especially commercial partners. Liliana [37 years old], a sex worker, commented, "*When they asked me*, *’Without a condom*?*’ I did not accept providing the service*." Participants also stated that they were aware that, for example, the use of drugs or alcohol could compromise their skills to use condoms.

Participants identified routine testing for HIV as an additional prevention tool they can use to manage their risk of HIV. For instance, participants mentioned seeking testing to ensure they are negative after a risky encounter or to continue to feel safe in a stable relationship if not using condoms. As Isabel [45 years old] mentioned:

*It is something I do every year [HIV testing] after stopping a relationship with my partner because I always have the risk [of contracting HIV]*, *I always have it*, *and I have lived with this risk*. *[Isabel*, *45 years old*]

Among participants seeking femininization, very few in this sample, routine HIV testing was required as part of this process. This was the case for Catalina:

*I went to do the tests to get the hormones*, *I have to go many times to the EPS [the clinic]*, *get seen by a psychologist and a psychiatrist*, *and I have to go with the HIV test results*. *The test is done here [a community site]… this has given me a sense of relief*. *[Catalina*, *22 years old*]

Few participants reported low knowledge of HIV testing concerning both its importance and accuracy.

*I said*, *"I’m going to do it*.*" But in some way*, *I did not feel anything strange*, *and I said*, *"I am going with God*.*" And I did that*, *and everything was fine*. *So*, *I wanted to know if those tests are actually reliable*. *I think so*, *right*? *[Claudia*, *32 years old*]

*1.2. Knowledge and skills for PrEP*. Few participants were aware of PrEP or knew about PrEP in any depth. For instance, Isabel, a TGW leader with substantial experience in HIV activism, showed a surprising level of uncertainty in her knowledge:

*I’ve heard about it [regarding PrEP] very recently*, *this is new*, *right*? *I heard that it was a medication that allow people to be with a person living with HIV and the person did not get infected*, *right*? *I think that was what I’ve heard*. *[Isabel*, *45 years old*]

Of the few participants who reported being aware of PrEP, they stated they gained their knowledge through a friend, participation in the demonstration study conducted by the Pan-American Health Organization, or international media and online searches. The latter was the case of Brenda, who found PrEP information 5 years earlier but had not been able to access it yet at the time of the interview:

*Well*, *I remember that the first contact*, *that is*, *the first time I read the word "PrEP*,*" was an article from the BBC*, *I don’t know what I was looking for on the Internet and it was in a headline and it caught my attention … It was talking about how*, *in England*, *they were implementing PrEP and that this treatment was for prevention and that you took a pill*, *…*. *you took a daily pill*, *and you could prevent the transmission of the virus*, *so I was like*, *"Wow*,*" this is too revolutionary*, *and I remember that I loved the idea*, *and I remember that I did discuss it with a couple of friends*. *This was about 5 years ago*. *[Brenda*, *30 years old*]

Concerning skills related to PrEP, most participants mentioned they would maintain the use of condoms if they decided to start PrEP. Their reasons were related to the importance that condoms have had in their prevention of HIV, the high risk of HIV in commercial sex, and because PrEP does not protect against other sexually transmitted diseases.

*You know that PrEP is only useful for HIV*, *but you can also get syphilis or a papilloma [HPV]… Even if I take PrEP*, *I must continue using a condom*. *Yes*, *if I’m going to be with a client or with a man I haven’t met before*, *with new sexual partners*. *[Samanta*, *30 years old*]

For Fernanda, the effectiveness of condoms makes the use of PrEP for prevention less appealing.

*I’m not going to say I’m going to take this pill because I’m going to have sex and I’m not going to use a condom*. *No*, *I prefer to fuck and use a condom*, *but if suddenly I have an accident*, *I take the PrEP*. *[Fernanda*, *50 years old*]

Many participants also referred to their own ability to adhere to medications and do the monitoring, including doing HIV tests, and attend medical check-ups, as in this narrative:

*I would do anything to get it [referring to PrEP]*. *… To already have it in my hands and take it daily and go for check-ups every 3 months*, *as you say*, *and not stop using condoms*, *even if I take PrEP*. *[Liliana*, *37 years old*]

One participant reported having an aversion to taking pills:

*If at a given moment I want to be with someone and I don’t want to put a condom on*, *it doesn’t matter because I’m sure I’m not going to get it [HIV] because I’m taking some medicine*. *There would be the cool piece that you’re sure you’re not getting a risk*, *but the price you have to pay [laughs] would be eating all those pills … like it’s not my thing*, *it’s not my thing*. *[Isabel*, *45 years old*]

Participants mentioned that there is a need to educate TGW in the following areas: PrEP and hormones, the importance of condom use along with PrEP, the inability of PrEP to prevent infections other than HIV, how evidence on PrEP is generated, and the experiences of people using PrEP.

#### 2. Opportunities

This domain covered aspects related to how the social and physical environments can either enable or hinder the use of HIV prevention tools, including PrEP. The narratives in this domain include themes related to interactions with family, partners, and healthcare professionals and the discrimination and limited social opportunities experienced within these interactions.

*2*.*1*. *Pressure and support from family*. Physical, psychological, and sexual violence perpetrated by family members were narratives frequently reported. Participants also mentioned experiences of sexual abuse and forced sex at early ages as well as discrimination from parents because of their sexual orientation and search for gender transition. One of the most significant narratives came from Pilar, who experienced different types of abuse since childhood.

*I lived with my stepmother; my mother abandoned me when I was 4 years old*, *and the one who raised me was my stepmother until I was 18 years old*. *After those 18 years*, *I couldn’t stand them anymore*, *believe me*, *I couldn’t stand any more physical and verbal abuse*. *[Pilar*, *29 years old*]

For Pilar, those experiences led to social isolation, depression, and suicide attempts. She mentioned that those experiences have prone her to know more about her risk of adquiring HIV and avoid risky situations such as commercial sex. Others described having supportive families with "open minds" as a form of "good luck." For instance, Monica related how her mother taught her to use condoms when she found out her sexual inclination [not disclosed here], and Samanta described how her family approved her use of PrEP.

*My family knows that I have always worked [as an activist] on sexual health issues*. *Everyone in my house*, *even my cat*, *is sensitized to HIV issues and prevention*… . *I have tested all my sisters-in-law*, *and when I told them about PrEP*, *they said*, *"That’s so cool*!*" [Samanta*, *30 years old*]

The participants indicated that a lack of support from family members would affect the use of PrEP by TGW and increase experiences of discrimination. Participants felt that family members might think that the use of PrEP would lead to a more promiscuous life or that they are already living with HIV.

*There are people who hide it from their family so as not to trigger direct discrimination because the family may think that PrEP means HIV treatment*, *so they would conclude they have HIV*. *[Margarita*, *34 years old*]

Similar to Margarita, Brenda also reported that many TGW have difficulties with their families accepting the use of PrEP, and they attributed this to the families’ lack of information and HIV stigma.

*If they find out that a member of their family or a friend is taking PrEP and if they ask about it*, *and they received an explanation about PrEP*, *they would look at you with their stigma lens*… *and would think that person has HIV and is going to transmit it to me …*. *people think that way because they do not know how it is transmitted*, *they do not know how it works*, *and so this can increase the prejudices and the discrimination*, *in the workplace and also in the family*. *[Brenda*, *30 years old*]

Brenda also mentioned that this type of problem would not happen with her friends, especially among those who are also transgender.

*2*.*2*. *Pressure and support from sexual partners*. Commercial, regular, and casual partners also exert differential pressures concerning HIV prevention behaviour as described by the participants. In the case of having commercial partners, condom use is the norm; nevertheless, participants mentioned that for many TGW, their poor economic conditions can compromise their ability to persuade their clients to use condoms. Participants who perform sex work consistently reported that commercial partners offer a higher payment for the service in exchange for omitting condom use. In this regard, Margarita said:

*In sex work*, *some clients ask for sexual acts not to be performed with a condom*, *clients who offer them two*, *three*, *or four times the rate [for this]*…, *and many agree to them not wearing a condom to get a bit more money*. *Although there are many of us with greater awareness who challenge them [other sex workers] with not doing sex without a condom because there may be something suspicious in a person [client] and that can be risky for them*. *[Margarita*, *34 years old*]

Margarita continued her narrative by proposing that when practising condomless sex, TGW may use PrEP as an alternative to prevent HIV infection because other sexually transmitted diseases can be treated and eliminated unlike HIV, which is a lifelong infection.

Participants distinguished between the types of protection and measures taken when they have a regular partner vs a casual or commercial partner. Gabriela, as well as other participants, did not use protection with their stable partners.

*There came a time when we stopped using condoms*… *he was my partner*, *my husband … there is no possibility of using prevention*, *one is not able to prevent anything … it’s like*, *"He’s my partner and so I don’t take care of myself*.*" … in my case*, *I’ve always lived that way*. *[Gabriela*, *23 years old*]

However, participants also described being aware that not using condoms in stable relations is problematic, as they believed that many of the HIV cases among TGW were the result of transmission by their stable partners rather than their commercial partners. As Samanta [30 years old] explained, *"Many transgender have had it [HIV]*, *I believe 50% have been infected by their [regular] partners and not by their clients*.*"* Given this belief, some participants stated the use of PrEP was an important addition to HIV prevention.

*2*.*3 Peer interactions*. Contrary to the largely negative influence of partners and family, participants have established good relationships with peer leaders in their communities. Through their interactions, they have acquired knowledge and skills for HIV prevention.

*About 2 years ago*, *some transgender women and I were invited to an event at the centre*… *There they taught us a lot of things about HIV*, *prevention*, *and all that*. *They explicitly taught us how to put a condom on a plastic penis*. *[Catalina*, *22 years old*]

According to narratives, organizations and transgender peers increase access to HIV testing and condoms and, more importantly, they interact with transgender populations in their places of residence and work. This latter benefit is vitally important to TGW who work in the streets, as they may have fewer economic resources, less time, and difficult schedules that are not compatible with prevention tools offered by the health system. The narratives of Mariana and Denise are examples of these positive interactions:

*I have a trans friend in an organization*, *and she contacts us every time they are going to do these tests or training*… *she does not hesitate to tell us and so far*, *I have always been there every single time they do them*. *[Mariana*, *44 years old*]

*A friend of mine [who works at a community-based organization] sometimes gives them [referring to condoms] to me for free*, *so I do not need to buy condoms*. *Although I know using them [condoms] is good for my health … I rather buy Colgate [toothpaste]*, *buy myself something else*, *because times are tough now*. *[Denise*, *24 years old*]

Participants felt that their peers are like their sisters and that they will support them if they take PrEP, as in the case of Catalina:

*If I told my friend about it [referring to PrEP]*, *she would say yes*, *she is a friend who has supported me in many ways; she would tell me*, *"No*, *obviously yes*, *obviously so you can be protected*.*" She always tells me the same*, *"You know that you can hardly trust these men*.*" That is*, *if you have a good time*, *that’s good*, *but rather you protect yourself more and feel peaceful*. *She thinks the same as I do*. *[Catalina 22 years old*]

*2*.*4*. *Health system interactions*. Although narratives regarding interactions with the healthcare system were diverse, the majority were related to negative interactions with healthcare professionals. The narratives detail experiences of stigma, discrimination, and lack of knowledge on the part of healthcare providers. For instance, participants described how providers often associated transgender identity with HIV; consequently, any health problems they reported were assumed to be HIV-related.

*The first thing they do is an HIV test because there is still the stigma of HIV since we are trans women and we are classified as sex workers; they will always test us for HIV even if we do not have any symptoms*. *[Margarita*, *34 years old*]

Participants also mentioned a lack of knowledge on transgender care and acceptance of gender identity among healthcare workers, as presented in the following narratives.

*I would say that most of them [experiences with the healthcare system] have been frustrating because I have encountered medical professionals who do not know enough about the subject [referring to sexual transition]*, *and we would like them to be people who are prepared and who know about such subject*, *who know how to offer care when a trans person seeks health services*. *I felt discriminated against*. *[Brenda 30 years old*]

*The last time I went to the [hospital]*, *a young man*, *this happened with men more than with women*, *the young man treated me as if I were a man*, *and I said*, *"I am a trans" and he said*, *"But you’re still a man*.*" He told me like that*, *so I felt bad*. *Do you understand me*? *[Pilar*, *29 years old*]

Some of the interactions with healthcare professionals were extremely distressing. This was the situation for Ariana, whose care was refused. She explained, "*The doctor who treated me felt disgusted when he saw me*, *I don’t know*, *he didn’t want to assess me*, *he asked few things and that’s it*, *he took me out of the office*.*" [Ariana*, *34 years old]*

Of the few participants aware of PrEP, they mentioned that healthcare workers do not know about it. Margarita advances an explanation for this that the lack of knowledge among healthcare workers is related to HIV stigma and likely also a stigma against the use of PrEP.

*Yes*, *I have asked them [about PrEP]*, *but they cannot*, *well*, *those that I have asked don’t seem able to address the problem directly and quickly because they also see it as a taboo*. *After all*, *they say "no*,*" or there are many who do not work on HIV because they are also afraid of it*, *or they don’t know about it … Among them*, *they also have a certain stigma*. *[Margarita*, *34 years old*]

Brenda thinks the negative experiences of many TGW with professionals will make it difficult for them to seek access to PrEP through the current healthcare system.

*Exactly … like having to constantly face that*, *I don’t know*, *that they make you undress*, *that they ask you questions*, *that they take blood from you*, *that they do all that to you*. *I would say*, *"Do not do that to me*.*" It doesn’t seem like an obstacle to me*, *but I know that many people do not like these routines*, *these types of periodic check-ups*. *[Brenda*, *30 years old*]

However, given the importance of monitoring, some participants felt that health services and professionals should be the ones who need to monitor PrEP. This was the experience of Samanta with PrEP.

*They could take control of who is taking it; if the patient is reacting positively*, *if they are taking it as prescribed*, *or if they are not taking it consistently*. *Then in the health system*, *it would be much better because the attending physician is there*, *and telling them that*, *"Yes*, *the treatment is doing a great job for your kidney or liver or [otherwise] stop it immediately*.*" Then it would be better using the [healthcare] system*, *I think it is better for your health*. *[Samanta*, *30 years old*]

In general, participants recommend more education and training for healthcare workers in HIV and transgender care.

*2*.*5*. *Cost and other types of access*. Like the negative interactions with professionals in the health system, participants reported problems with access to care and the need to file an appeal or sue the system to obtain the care they need.

*I had to file an appeal*… *so that they could grant me what I am rightfully entitled to*. *So*, *it is a tiring process*. *I feel that as a trans woman in a country like Colombia and with this system*… *the laws are not observed*, *so it is very complicated*. *[Margarita*, *34 years old*]

Some participants worry that the medications for PrEP are not consistently supplied, as they have had experiences with other conditions where supply was interrupted. They felt that having access to condoms or asking for an HIV test will take longer and will not be enough, which contrasts with what is provided in community organizations. Given the disadvantageous financial conditions of many of the participants, they also mentioned that PrEP needs to be offered free of cost in sites accessible to them and covered by health insurance plans.

*The obstacle is that they are a little expensive*… *because there are people who do not have the means*, *that would be an obstacle*. *Another [obstacle] would be that the EPS [the healthcare provider institution] would not provide them because they [TGW] are not affiliated [with their institution] or do not pay the co-payment*. *They should provide them for free*. *[Liliana*, *37 years old*]

*In a nearby place*, *because we often don’t have access to money to pay for transportation*, *a bus*, *a motorcycle*, *a taxi*. *Many times we don’t have that money … because we have [other] responsibilities*, *such as paying rent*, *buying food*, *pay bills*, *and often we don’t have it*. *[Gabriela 23 years old*]

Regarding these obstacles, participants suggested different options for PrEP delivery, such as having PrEP available in a nearby pharmacy, community organizations, or even home delivery. Samanta, who takes PrEP, recognizes that once the demonstration project finishes, she will have difficulties accessing PrEP.

*I wish that would happen*, *that I would go to my health system and request the PrEP and continue taking it because I know that once this project ends [referring to the demonstration project]*, *that will be it*, *and that is bad*. *[Samanta*, *30 years old*]

*2*.*6 Community interactions and opportunities*. TGW experience different forms of discrimination: against their identities as TGW, their occupation as sex workers, their HIV-positive status, or their poor socioeconomic condition. For instance, Margarita mentioned increased levels of violence toward TGW perpetrated by different community actors:

*I don’t do sex work*, *but if I’m with girls and I approach girls who do sex work*, *many of them are raped*, *raped with a knife*, *raped because they live on the street*, *raped because they expose themselves to certain social problems*. *Even with the police and with the environment where they live*, *and in certain types of situations*, *there have been*, *let’s say*, *injuries*. *[Margarita*, *34 years old*]

Participants also mentioned how those experiences become obstacles to education, employment, housing, appropriate access to care, and the use of preventive measures.

*That is very true*, *many people*, *especially trans girls*, *often do not have the chance to study and end up choosing prostitution because it is the path that society offered us*. *Or [there are] some [transgender] girls who have studied because they received support and they were able to move on*. *But many of us have a hard life*, *and we don’t have a way to do that [referring to study and work]*. *[Gabriela*, *23 years old*]

Participants mentioned that within the TGW population, some groups are more vulnerable to discrimination than others. The participants cited those with lower socioeconomic status, those who do sex work, and Afrodescendants.

*There are different problems that a single person would need to handle*, *the burden is 10 times heavier than that I have to bear*. *I live in a stratum-3 or -4 house [socioeconomic stratum]*, *I am White*, *I’m not Black*, *and*, *basically*, *I live with all the privileges that those girls don’t have*, *and they have to fight day by day*, *stealing*, *trying to earn their daily living whoring in the streets*, *in order to survive … and trying to have a level of life that they would never reach because they wanted to be happy but their families do not accept them*. *[Margarita*, *34 years old*]

Participants consider that the HIV stigma that persists in society may become a barrier to accessing PrEP, as taking PrEP may be perceived as them having HIV.

*Well*, *it will make me feel a little bit ashamed*… *if you go somewhere and there are people … associated with having HIV and they realize that [and say]*, *"Look*, *she is coming here to get that*.*" You know how people are here in Colombia*. *They are going to think that I have HIV*, *I mean*, *people will think that if you are taking a pill that is for HIV*, *they will think that you have it*. *[Liliana*, *37 years old*]

Participants felt that the fact that TGW have always been at high risk of HIV has perpetuated the stigma against them. They suggest that PrEP-related messages should also be focused on the non-LGTB population, as heterosexual men and women are also at risk of transmission and acquisition of HIV.

#### 3. Motivation

This domain summarizes the narratives of the participants related to their perceptions about PrEP, their intentions to use it, the reasons to use it, and the possible psychosocial barriers to their use. The narratives of the TGW demonstrated their keen interest in using PrEP.

*3*.*1*. *Reduce the risk and fear of HIV*. Most of the participants perceived themselves as having a high risk of contracting HIV. They also recognized that their sexual behaviours increase their risk. Many reported being aware that TGW are at higher risk of HIV in general either because they know the statistics or because they have family and friends with HIV. For participants, this is translated into constant fear of becoming HIV-infected and suffering the consequences of the severe stigma they anticipated they would face.

*It is something hard for a person having to take pills from the time they get up to the time they go to bed*, *knowing that if they live with their family*, *they have to have everything separate*, *and they will always be named as having it do it like [unintelligible word] … you know*, *like saying*, *"No*, *he has HIV*, *he has to stay separate from us*.*" "No*, *he’s like a plague*.*" "Use the gloves*, *he has HIV*.*" It’s something very hard*, *I think*. *[Gabriella*, *23 years old*]

For most participants, their primary motivation to use PrEP was related to the possibility of reducing HIV risk, either personally or in their communities. Monica’s and Gabriela’s narratives covered both the personal and community benefits of PrEP, such as less fear of being infected, gaining confidence in being capable of preventing HIV infections, gaining control of decisions on prevention, and reducing the mental health consequences of a diagnosis of HIV in a context of significant discrimination.

*Yes*, *there would give me peace of mind*, *with PrEP yes*. *So*, *I wouldn’t go with so much fear to take the test*, *you know*, *that being positive is something that will be difficult to hear*, *that somebody tells you*, *"You are positive*.*" Although we cannot rule out it can happen*. *[Monica*, *22 years old*]

*I think we are going to see more self-confidence*, *that we are going to be able to prevent this issue of HIV*, *spreading it … and the negative part of PrEP would be like not knowing what adverse effects can have on the body*, *to our health*. *[Monica*, *22 years old*]

*Well*, *I think the positive thing is that it would prevent the disease*, *and it would stop many deaths and many suicides because most of the suicides of homosexuals or trans girls come from this disease*. *[Gabriela*, *23 years old*]

*Of course*, *for me*, *yes*, *some girls are afraid of that [HIV infection]*. *But for me*, *that is essential [prevention of HIV]*. *I think that*, *as a trans woman*, *one is exposed to many things; one is exposed to sex*, *one is exposed to sleeping with men you do not know*. *Yes*, *I can tell you … that I would do it [use PrEP]*, *of course*, *I would do it*. *For me*, *it would be a control*, *a check-up*, *to know that I am healthy*. *[Gabriela*, *23 years old*]

*3*.*2*. *Concerns about the use of PrEP*. Side effects, unknown efficacy, interference with hormone treatment, and how the use of PrEP medications may affect other medical conditions were among the concerns reported. However, none of those concerns seem to disfavour its use among the participants.

*Negative points would be the side effects*… *that may arise*, *for example*, *I don’t know if there are enough studies that show what the implications are of taking this medication for ’x’ time*. *[Brenda*, *30 years old*]

*What happens in your body*? *Has that been studied or not*? *What happens if I start taking PrEP and I see that it’s affecting my hormone treatment in some way*… *that would be a reason for people to say*, *"Oh no*!*" [Margarita*, *34 years old*]

One participant expressed concern about the possibility that people could counterfeit PrEP pills and sell them and that the counterfeit pills would not effective or they could be combined with illicit drugs. Regarding the first point, Margarita mentioned, "*Colombia is practically the country that counterfeits most things in the world*, *and this type of pill or treatment may also be easy to fake*, *and that could be a problem*.*"*

*3*.*3*. *Mental health and emotional states*. Narratives of participants mentioned how following the hormonal transformation of their bodies and the various expressions of social discrimination affect them physically and emotionally. Participants mentioned suffering from depression and anxiety and having suicidal thoughts. Pilar commented on her experience with mental health issues as follows:

*It has been a very strong process [acceptance of her transsexuality]*, *too strong*, *they are very strong processes*. *If I had chosen that life would have not been like this*, *I would have preferred to have died because it is a very hard process*, *where you see it is a very hard process because now I have had anxiety*, *depression*, *very strong obsessive disorders*. *So*, *I think so*. *[Pilar*, *29 years old*]

Drug and alcohol use and abuse among TGW was also identified as a consequence of the persistent fear and discrimination TGW living with HIV experience. As mentioned by Gabriela:

*Many times*, *suicide*, *other times*, *they began to consume more drugs than usual*. *They began to consume more alcohol than usual*, *and that makes death accelerate faster because*, *well*, *you know that the defences get affected and*, *then they accelerated death faster*. *[Gabriela*, *23 years old*]

The participants indicated that substance use and abuse would influence the intention of TGW to use PrEP negatively, such as decreased adherence or interest in taking additional pills when not sick. Gabriela and Samanta explained:

*To calm my sadness*, *I used to take everything [drugs]*. *Then I became so unhealthy because I neglected my hormones and my thyroid*. *I suffered from insomnia*, *and I began to attend a drug addiction program*. *There*, *they recommend us not to take anything*, *so we don’t get addicted*. *[Gabriela*, *23 years old*]

*Other people could be discouraged about taking PrEP because of a state of mind*, *such as the girl does suffer from depression or psychiatric problems … sentimental issues would be what would suddenly lead a trans woman to turn on not taking it*. *[Samanta*, *30 years old*]

Other participants’ narratives reflect high self-esteem and resilience among TGW, aspects that could favour their use of preventive strategies, including PrEP. As Samanta, who uses PrEP, described:

*The motivation came from ourselves too*, *of wanting to do it*, *because I don’t need someone else to support me to do something I want*, *right*? *Let’s say if my family would have opposed me taking it*, *I do it anyway*. *[Samanta*, *30 years old*]

The importance of having a good mindset to be willing to use PrEP was also mentioned by Catalina:

*So*, *it is more a self-commitment*, *a commitment to you*, *to what you are doing and your health*. *That is*, *if you do not take care of your health*, *nobody is going to take care of it*. *That is the reality*. *Unless it is your mother*, *well*, *that is reality*. *[Catalina*, *22 years old*]

### Mapping with COM-B

Overall, nine barriers to PrEP uptake were identified [**Table 4**]. Most of the barriers were observed in the opportunity domain, where negative interactions with family, partners, and healthcare professionals may limit PrEP use and access. The cost and lack of adequacy and accessibility of the current health service are also likely barriers to PrEP access and continuity of usage. Community opportunities reinforce the HIV stigma and structural barriers faced by TGW, which may influence the place and type of services TGW need. The context of sex work may also limit access to and use of PrEP as well as the use of prevention tools [e.g., condoms]. In contrast, fewer obstacles were found in the capability domain, where low PrEP awareness and knowledge may limit PrEP use. In the motivation domain, mental and emotional states and side effect concerns may act as important barriers. Using the BCW [**Table 4**], we can derive interventions to address barriers as follows: At the intervention function level, our set of interventions includes the provision of education and training, the use of interventions related to enablement, environmental structural changes, and persuasion interventions. At the policy level, we identified the need for communication campaigns, changes in service provision, and legislation on sexual rights. The discussion section will provide an extension of the possible interventions.

**Table 4:** Summary of barriers mapped using COM-B and possible intervention functions and policies to address barriers, BCW.

Which barrier needs to be addressed?	How may it relate to PrEP uptake?	In which COM-B domain does the barrier operate?	Intervention functions to address the barrier	Policy options for addressing the barrier
1-PrEP knowledge and perceptions.	Low awareness and concerns that may limit acceptance and adherence	Capability	Education and training	Social media marketing and communication strategies
2-Family relations	This can be an obstacle to using PrEP due to fear of HIV stigma; poor support and more violence that led to negative emotional states	Social opportunity and Motivation	Enablement interventions	Legislation to protect TGW sexual rights
3-Sexual work environment	Lack of power to negotiate condoms in the setting of financial hardship; more experiences of stigma, and barriers to access preventive services’ lack of social opportunities in terms of education and income	Social opportunity, and capability	Enablement, and environmental structure interventions	Legislation to protect TGW sexual rights
4-Stable Partner relations	This can be an obstacle to using condoms and PrEP because of lack of support or experiences of safety and trust that decrease use of preventive tools.	Social opportunity	Persuasion and training interventions	Legislation to protect TGW sexual rights
5-Interactions with healthcare workers	The lack of knowledge and level of stigma decrease access and opportunity for PrEP discussions	Social and physical opportunity	Environmental structure and Training interventions	Change service provision, guidelines
6-Health service provision	Limited access because of discrimination and fees for services. Affecting PrEP adherence because of barriers to navigation of the system and medication provision	Physical opportunity	Environmental structure intervention	Change service provision and Legislation to protect TGW sexual rights
7- Community interactions and opportunities	Can be an obstacle for access to and disclosing the use of PrEP; limit social opportunities, increase levels of discrimination	Social and physical opportunity	Enablement, and environmental structure interventions	Legislation to protect TGW sexual rights
8- Mental and emotional state	May limit adherence and interest in taking pills	Capability and motivation	Enablement, and environmental structure interventions	Change service provision:
9- Concerns about side effects	May limit adherence and interest in taking pills	Capability and motivation	Education and training; persuasion	Guidelines and Change service provision:

## Discussion

### Main findings

Through this work, we have first identified key barriers to PrEP uptake by TGW in Colombia: low PrEP awareness and knowledge [capability]; negative relationships with partners, family, and health services, which are stigmatizing, violent, and result in social exclusion [opportunities]; and psychosocial factors related to mental health issues and side effect concerns [motivations]. Second, we have identified important facilitators for PrEP uptake: good knowledge and skills to use condoms and other HIV preventive measures [capability], positive interactions with community leaders and organizations [social opportunities], and a strong motivation to decrease the risk of HIV acquisition [motivation]. Lastly, we mapped the barriers identified in the narratives with the recommended interventions and policies in each COM-B domain. Overall, our results highlight the need to develop interventions and policies that 1] educate and train TGW, 2] increase PrEP awareness in the general public, 3] increase community support to facilitate uptake and adherence, 4] improve and integrate the provision of prevention services and care for TGW, 5] increase PrEP awareness and knowledge among health care professionals, 6] facilitate the coverage and accessibility of PrEP in the health system, and 7] reinforce/improve the current legislation to protect the sexual rights of TGW with special emphasis in those who perform sex work.

### Comparison with other studies

Our results are consistent with those of other PrEP studies with TGW and are comparable with our literature review reported in **Table 1**. Capability factors, such as low awareness and poor knowledge of PrEP among TGW, have been reported in other countries [21]. The level of PrEP awareness found among TGW in Colombia [24] was lower than that of TGW in Brazil and Peru [26–28]. In Colombia, the low awareness of PrEP among TGW has been related to low levels of education and socioeconomic status [24], and likely related to low access to the Internet, where information on PrEP has been widely disseminated [18, 29]. This contrasts with the knowledge and use of other preventive tools for HIV prevention, including a positive perception among TGW of condom use and routine HIV testing [3]. This knowledge was attributed by our participants to the efforts of peers and community organizations in increasing access to condoms and regular HIV testing campaigns. Although it is well known that knowledge of PrEP may increase intention to use PrEP [6], the low awareness of PrEP found in our participants did not directly translate into a low willingness to use PrEP. In a previous analysis of our survey data, 67% of respondents reported an intention to use PrEP [24], and no relationship was found between intention to use and PrEP awareness and knowledge [24]. This discrepancy [between knowledge and intention] has been described in other populations [6, 9, 30] relates to motivational factors, specifically, a perception of being at high risk of contracting HIV and the possibility of controlling this risk during sexual encounters. Such motivational factors were also expressed by the sample of TGW in Colombia, whose main motivation to take PrEP was the fear of becoming HIV-infected.

Similar to other studies, the aspects related to the social opportunity domain were the most relevant to understanding possible obstacles to PrEP uptake [8]. The lack of positive social relationships [family and partners] and especially the violence TWG experience within these relationships, is prevalent in Colombia and other Latin American settings [31–34]. Negative interactions with family and partners constituted key barriers to HIV prevention, including PrEP, mainly through their effects on mental health, the use of psychoactive substances, and low condom use [3, 35, 36]. In this sample, these interactions took the form of exerted violence, low access to material resources [e.g., income, employment, and education], and discriminatory experiences when accessing healthcare. The context of sex work seems to be the intersection where all these negative forces combine and the effects are dual and paradoxical: on one hand, the perception of high risk of HIV acquisition seems to favour PrEP uptake, but on the other hand, the negative experiences are detrimental for PrEP access, adherence, and persistence [21].

Negative interactions with health services and their providers were also common narratives from the participants, which are comparable with other contexts, and would likely discourage PrEP uptake and adherence [21]. The experiences reported by TGW in our study prompt us to infer that sexual health education that is inclusive of gender diversity is lacking for Colombian healthcare providers. Emerging data suggest that HIV prevention and healthcare services are more acceptable to transgender people when they are de-stigmatizing and include access to gender-affirming care. In our study, transgender populations face challenges due to gender and HIV-related stigma, unmet gender-affirming needs, and inadequately trained healthcare providers. Similar barriers were described by MSM and HIV care professionals working in HIV clinics in Colombia [16, 18], underscoring that provider education requires priority attention [37].

Concerns about possible side effects of PrEP and its interactions with hormonal therapies were motivational obstacles in our population and have been reported elsewhere [19, 30, 38–40]. Narratives of participants in the ImPrEP project in Mexico show how these factors related to a lack of adherence to PrEP over time [41]. However, of the motivational factors, the most relevant for PrEP uptake in TGW seem to be the presence of poor emotional states and the use of psychoactive drugs. It is well recognized that poor mental health and the use of drugs and alcohol are mechanisms TGW use to cope with violence, stress, and lifelong structural discrimination [3, 42, 43]. Studies have shown that poor mental health and abuse of substances translate into less use of HIV preventive services and lower levels of PrEP awareness and adherence [43]. Addressing the needs of and providing effective support to TGW during their transition must consist of gender-affirming healthcare linked with appropriate mental health services, which is greatly needed in the Colombian context.

Conversely, our participants reported consistent positive interactions with community leaders. Interactions with transgender leaders have been demonstrated to positively influence access to and use of HIV prevention tools [44], including PrEP. Community organizations in Colombia are recognized for their leadership and advocacy for transgender rights and their work in HIV prevention through training, condom distribution, and screening [45]. These efforts result in positive perceptions regarding increased condom use and the use of preventive services, as seen in the narratives of our participants. In the case of PrEP, different studies with TGW have shown how peers can fill the gap in information, counselling, and navigation to access and adhere to PrEP [46, 47]. The integration of community organization experiences with PrEP provision into the healthcare service has been one of the most important recommendations from healthcare practitioners and communities, as described in our previously published work [16] and supported by other studies [18, 48, 49].

### Expanding possible interventions

According to the barriers identified, the first intervention function to ensure the uptake of PrEP should aim to increase awareness through education focused on providing adequate knowledge [education and skills training] most relevant to TGW. Specifically, the educational content must include tools for TGW that increase their communication skills, especially when dealing with health professionals, reinforce and provide language for condom negotiation [e.g., with commercial partners], and information related to PrEP side effects and interactions with hormones to improve confidence in using PrEP. This can be achieved by training transgender community leaders who can promote access to PrEP information for those who need it, near the areas where potential users live and work, with messages tailored to their needs, beliefs, educational levels, and preferences. Additionally, engaging TGW who have already used PrEP could serve as a persuasion strategy to increase confidence in the safety of PrEP medications.

The second intervention function is related to "enabling intervention." In this case, the intervention should aim to increase support and resources to access, uptake, and sustainable use of PrEP. One potential intervention is counselling sessions before and during the use of PrEP. The counselling must ideally be delivered by community and healthcare organizations in conjunction with healthcare providers [43]. Motivational interviewing and other psychological interventions could be adapted and used to provide this type of support. Another promising intervention is training TGW to become PrEP champions and/or PrEP navigators. PrEP champions/navigators can guide TGW in accessing PrEP, help in navigating the healthcare system, advocate for PrEP access, and offer support on medication adherence. Existing navigator interventions could be adapted or new programs developed specifically for the Colombian context [50, 51]. Lastly, support groups operating in community centres or clinics can increase social and emotional support and foster a sense of community [especially among those more marginalized and discriminated against], which can enhance engagement in care and improve mental health.

The third type of intervention function resulting from our study relates to "environmental structure change," which in our case, means changing the physical and social context to facilitate accessibility, acceptability, and adherence to PrEP. This may include 1] integrating the provision of PrEP services into community organizations and sexual health clinics [as implemented in the demonstration study in Colombia], encompassing health insurance navigation, healthcare system navigation [referral to PrEP providers], and substance use and mental health support. Examples of this type of program have been implemented with positive outcomes for TGW [52]. 2] The integration of gender-based violence programs into HIV prevention programs to address the multiple experiences of violence of TGW, especially sex workers [31, 43]. 3] Training of healthcare providers through the development and application of a curriculum for primary care providers on HIV prevention and care, including PrEP, a basic understanding of trans healthcare, and strategies to enhance gender-affirming care [53].

At the policy level, opportunities exist to decrease TGW’s vulnerability and increase their access to gender-sensitive programs [see **Table 1**]. Given TGW’s low PrEP awareness and their concerns about its use, the first policy should aim to increase awareness through campaigns containing appropriate messaging that is culture- and gender-sensitive and context-specific [54]. The stigma must be addressed with communication and education that adheres to accurate information, uses appropriate language, and avoids the misrepresentation of people living with HIV. A second policy should promote regulations and legislation that effectively protect the rights of TGW and sex workers, especially their right to sexual health. This legislation should include a gender-affirming approach to care that prioritizes prevention over cure and reduces the systemic discrimination of TGW in terms of access to and quality of healthcare. The third policy relates to changes in health service provision, namely innovative ways to offer services that are acceptable and geographically accessible to TGW. Examples of these changes may include the integration of services as mentioned above, the training of nurses or community health workers as additional providers of PrEP services, integrating peer navigators as providers of some PrEP services, and creating mechanisms to provide PrEP free of cost. The experiences of other Latin American countries with more advanced work on PrEP, such as Brazil, may inform the provision of services and the financing of PrEP in Colombia [55]. Notably, studies elsewhere have demonstrated that healthcare insurance is related to increased PrEP uptake by TGW [56, 57].

### Strengths and limitations of the study

The fact that recruitment was only possible through community venues allowed for the inclusion of the most vulnerable populations who could not be reached by social media, a recruitment strategy that has been successful for studies with MSM [58]. Despite the possible limitations in the sample, our results are consistent with other studies and go beyond solely offering a description of barriers and facilitators by adding a mapping exercise to identify possible solutions. Although this mapping offers a partial perspective, it is supported by other types of analysis conducted by our group [25] and aligns with recommendations from other publications [59, 60].

## Conclusions

This study has identified a series of barriers and facilitators that influence the uptake of PrEP by TGW in Colombia. These findings highlight the importance of addressing not only the limited access to PrEP in the current Colombia health system but also the social and psychological factors that impact decision-making related to HIV prevention among TGW in Colombia. Identified barriers, such as poor knowledge about PrEP, stigmatization in the family and partner environment, and a lack of support and care from health services, should be addressed by implementing educational and awareness programs. This would help improve TGW’s information and understanding of PrEP as well as promote healthy and stigma-free relationships. On the other hand, the facilitators found, such as knowledge about condom use and other HIV preventive measures, positive interaction with community leaders and organizations, and motivation to reduce risk, can be leveraged to promote acceptance of PrEP. It is important to strengthen these facilitating factors through interventions that foster the empowerment and active participation of TGW in decision-making related to their health. Overall, this study highlights the importance of adopting a comprehensive, multidimensional approach to address barriers and facilitators to PrEP acceptance among TGW. Briefly, our results indicate a need for the development and implementation of interventions and policies related to the following: 1] peer navigation, 2] integration of provision of prevention services and care for TGW, 3] creation of a curriculum for education and training on HIV and PrEP for health care professionals, 4] community-based delivery PrEP services, and 5] gender-affirming care, policies, and interventions. To successfully implement PrEP in the Colombian healthcare system, it is essential to engage TGW and transgender community organizations as key allies through their work as carriers of appropriate and effective messages, peers, and support resources.

## Supporting information

S1 Text Interview guide. (DOCX)
