# Supplementary material for: Barriers and facilitators to HIV pre-exposure prophylaxis uptake among transgender women in Colombia: A qualitative analysis using the COM-B model
Source: PLOS Glob Public Health. 2023 Sep 27;3(9):e0001395. doi: 10.1371/journal.pgph.0001395 (PMC10529613; doi:10.1371/journal.pgph.0001395)
Supplement: S1 Text — (DOCX) [file pgph.0001395.s001.docx]

**"DEVELOPMENT OF IMPLEMENTATION STRATEGIES FOR HIV PRE-EXPOSURE PROPHYLAXIS IN HIV CLINICS IN COLOMBIA: PROTOCOL OF A MIXED METHOD IMPLEMENTATION STUDY"**

**Interview Guide - Qualitative Interview Phase**

**Objective:** to explore the perspectives of HIV-negative transgender women in relation to HIV prevention and the role of PrEP in prevention (barriers and facilitators). The model of "social action theory" / social actino theory is used as a basic conceptual model

**Eligibility criteria:** transgender women, over the age of 18, who participated in the quantitative survey and who:

- **Be knowledgeable and express a desire to use PrEP.**
- **Have used PrEP or PEP.**
- **Have knowledge of PrEP, but don't want to use it.**
- **Have no knowledge or intentions of using PrEP**

**Before starting with the prevention questions, it is pertinent for me to consult the key informants a little about them:**

Who are you?

What do they do?

**Marital status or sentimental situation? (This allows me to inquire about sexual practices and methods of prevention)**

**GENERAL PREVENTION QUESTIONS - APPLY TO EVERYONE**

- What does "prevent" mean to you?
- What prevention strategies do you use to avoid the risk of HIV infections?
- What strategies are used by transgender women to avoid HIV infection?

Inquire as appropriate for specific strategies of transgender women suggestion: HIV testing, condoms, serological selection, etc. How easy or difficult is it for you to use condoms? get tested for HIV frequently? Talk about your sexual orientation?

- **How did [where/from whom] did you learn these prevention strategies?** (Suggestion: peers, friends, agencies, health care providers, social media, Internet, school, television, etc.)
- **How effective or ineffective do you feel the different HIV prevention strategies are?** (Hint: What inspires that confidence? What doesn't inspire that confidence? What makes you feel more confident about its effectiveness?)
- **How confident are you that you won't have HIV if you continue to have the same sex life you have so far? (**Suggestion Why 'do you feel this way’? What would make you feel safer?)
- **Do you have a plan to change your sexual health and prevent becoming infected with HIV?** (Suggestion: why' yes, why not? what is your plan? inquire if PrEP is part of this plan)

**FILTER QUESTION**

**IT IS IMPORTANT TO CONSULT ABOUT YOUR KNOWLEDGE IN PrEP before asking these two questions.**

- Have you used any of these prevention strategies – explain and ask about PrEP or PEP
- Would you consider using PrEP, PEP?

**BEFORE STARTING WITH THE FOLLOWING MODULE IT IS NECESSARY TO INQUIRE ABOUT THE CLOSENESS AND TRUST USED TO THE HEALTH SYSTEM-IPS**

1. How often do you visit the institutions providing health services?
2. Have you asked about your sexual health and rights?
3. How much information has health personnel given you? Do you consider it relevant, effective, and reliable?
4. What is your perception of health care professionals towards transgender women?

**FOR THOSE WHO KNOW ABOUT PrEP, BUT HAVE NEVER USED IT.**

- Have you ever asked or talked to your health care provider about PrEP? (Hint: Why not? Can you describe the process you used to ask about PrEP? Were you successful in obtaining information?; How much did your doctor know about your sex life before talking about PrEP? Was it you who asked about PrEP or was it your doctor?
- What was the experience of talking to a health care professional like about PrEP? (suggestions: ease, comfort, experience of the professional) - inquire about the particular case. What was that experience like? How much information did they give you? Quality of the information?
- What made you make the decision to ask about PrEP/ why do you think the professional suggested PrEP? (suggestion: Did you know other users? What was your relationship with institutions and workers in the field? because of your risk situations, because you like PrEP?)

**Ask about the background (Close Circle) and its relationship with PrEP. Where did you first hear about PrEP?**

- What aspect of this prevention strategy do you like best? (Suggestions: more sexual satisfaction, more prevention, more security...)
- What barriers or challenges would you face if you decided to use PrEP? (Tip: Who or what might be helpful? does your financial situation and/or health insurance coverage affect access to PEP and/or PrEP? explore family, partners, friends, and their perceptions)
- What barriers do you think your entire community may present? What can make it easier for you to use PrEP?
- How good would you be at taking a pill every day and getting checked every three months? (Tip: Who or what might be helpful? does your financial situation and/or health insurance coverage affect access to PEP and/or PrEP? explore family, partners, friends, and their perceptions).

**FOR THOSE WHO HAVE USED PrEP –PEP**

- What experience have you had with the use of this prevention strategy? (Positive impressions: pleasure, freedom, less anxiety) (Negative impressions: side effects, cost, stigma, adherence, access problems, substance use)
- What aspect of this prevention strategy do you like best? What were the best aspects of this prevention strategy? Which are/were the hardest/worst? How do they compare? (Hint: Has using these tools like PrEP/PEP affected other areas of your health? Does this have anything to do with your substance use?)
- What experiences, if any, have you had talking to your partners or potential partners (stable, sporadic, or commercial) about the use of PrEP? (hint: How did they react? What questions or concerns did you have? How did you guide them? Why didn't you talk to these couples about this? How was trust affected in your conversation?)
- How has your idea of "risky" and "protected" sex changed now that PrEP/PEP exists?
- **Has the use of PrEP/PEP changed your substance use, life, or sexual practices? (**Suggestions: The type of sex to have if you use or do not use condoms, the frequency of sex, the types of partners, and communication with partners and/or health care providers, whether you use substance when separating or having sex)

**FOR THOSE WHO MENTIONED BEING INTERESTED IN USING PrEP/ have never asked a professional**

- **Do you plan to use PrEP/PEP** in **the future, if it becomes available to you?**  (hint: (if you answer no, ask why not?) What would be the ideal way to access PrEP? What would be the ideal way to use PrEP? What kind of community support would be serious about using PrEP?)
- **What barriers or challenges would you face if you decided to use PrEP?** (Hint: Who or what might be helpful? does your financial situation and/or health insurance coverage affect access to PEP and/or PrEP?)
- **What or who might influence your decision to use (or not use) PrEP?** (hint: your partner, community support, friends? Other users?
- **What are the positive and negative implications of using PrEP)?** (hint: how do you think these might work for people who use psychoactive substances? Or for women who use hormones?)
- **Do you talk to others about PrEP**? (hint: Why not? Who do you talk to? How have you approached this topic? What were those conversations like? What/who will make you feel most about talking to other people about this? Where do you feel most about talking about this?)

**QUESTIONS FOR THOSE WHO ARE NOT INTERESTED IN USING PrEP**

- **Why wouldn't transgender women want to try PrEP?** (hint: Why' or why not? How does this affect the use of other prevention strategies? What are some of the reasons why people may not find PrEP acceptable??)
- **Why wouldn't you like PrEP as a prevention strategy?** (suggestion: Why' or why not? Explore knowledge, perceptions, perceived risk)
- **Do you talk to others about PrEP**? (hint: Why not? Who do you talk to? How have you approached this topic? What were those conversations like? What/who will make you feel most about talking to other people about this? Where will you feel with talking about this?)
- **What could change your interest in prEP use in the future?** (Suggestion: more information, more social support, access to care, etc.)
